# Morality and Sex

**Published:** 1914-10

**Authors:** 


### The Department of Eugenics, Mental and Social Hygiene

CONDUCTED BY
DR. MALONE DUGGAN
813-814 Gibbs Building, San Antonio, Texas
All communications for this department should be sent to DR. DUGGAN

### Morality and Sex.

Several months since we presented in this department an article from *Critic and Guide,* by Dr. Rutgers of The Hague, Holland, on the "Deliberate Control of Procreation." The idea was so well received that the author was requested to express his views on the question of the morality of sex. It is, therefore, with pleasure that we give below the doctor's very cordial response to this invitation. He says in full:

All honorable persons who have to educate young people are aware of the fact that the whole career of their pupils, when they become adults, can be at once spoiled by some caprice of their sex life. There is nothing that can bring to us so much trouble and despair. For that reason pedagogues are likely to hate all manifestations of sex life. And to look upon sexuality as the greatest immorality. Had *they* created man, surely they would have created him *sex-less,* just as, indeed, our fantasy has created angles sexless. Even the whole theory of good and evil has been based upon the sinfulness of the body and the holiness of abnegation. That is the reason why until now success in sex-education has been practically nil. Stemming the tide of living energy never will succeed. You may dam a river but you cannot stop its flow. We all know there are two fundamental stimulants of life,—the struggle for existence and sexuality. The former makes us "fighting," the latter, loving. Just so we must realize that all higher qualities such as altruism, sympathy and charity are due to the latter, even as our own existence is due to it.

Aware of the ill-success of moral education up until the present, we must now turn our attention to the other side; we must build up instead of pulling down. Sex reform is wanted only when we do not attend to physiologic and hygienic laws. When we do justice to our vital energy and normal aspirations we can avoid the abuses and excesses that indeed befall us from sex-life.

Unwanted pregnancy is one danger in sex-life. Originally this danger was the fundamental reason why sexual intercourse was stigmatized as being so immoral. Now that we can control this faculty making sexual intercourse sterile when desired we must revise our ethical code. Sex-morality formally based upon the brute fear of pregnancy, now must be built upon higher ethical principles, mutual respect and sympathy.

Venereal diseases are another danger. Just as with all contagious diseases, seclusion was the first method of prevention practiced, but the success attained always proved inimical. Then followed disinfection and now rational hygiene will clear up the whole situation.

An ancient danger in sex matters is the blindness of passion, the lack of self-control and the extravagance of debauchery. These are namely the result of our system of morality. Just as with pent-up explosive substances, suppression of sex feelings provocates the most terrible outbursts. The time is now coming when sex-life will be properly respected and conserved through conscious election. It is the flower of human life, and when flowering in the sunshine of life it will become a factor of happiness and the greatest blessing in our human existence.

